# Biological effects of two nano-composite resins on human gingival fibroblast (an *in vitro* study)

**DOI:** 10.1016/j.jobcr.2025.07.006

**Published:** 2025-07-16

**Authors:** Mai Gamal Mahmoud, Ali Shamaa, Noura Mohammed Bakr, Maha El Shahawy

**Affiliations:** a Department of Oral Biology, Faculty of Dentistry, Minia University, Minia, 61511, Egypt; b Oral Biology Department, Faculty of Dentistry, Kafrelsheikh University, Elgiesh street, Kafrelsheikh, 33516, Egypt; c Department of Oral and Dental Biology, Faculty of Dental Medicine for Girls, Al-Azhar University, Cairo, Egypt

**Keywords:** Human gingival fibroblast, Nano-composite resin, IL-1β, IL-6, Mode of cell death

## Abstract

**Objective:**

To elucidate the genotoxic and cytotoxic effects of two different nano-composite resins on primary human gingival fibroblasts (HGFs).

**Methods:**

The HGFs were isolated, characterized, and classified into 3 groups. The control group consisted of untreated HGFs, the Omnichroma extract treated HGFs (the OMN group), and the 3M Filtek Z350 xt extract treated HGFs (ESPE group). The cell viability, mode of cell death and expression of *interleukin-1β* (*IL-1β*) and *interleukin-6* (*IL****-****6*) were investigated after 72 h and 24 h of resin extracts' pre-incubation with HGF.

**Results:**

The isolated HGFs were characterized. The HGF viability was significantly higher in the OMN groups than ESPE groups at most of the concentrations. Total cell death was higher in the ESPE groups and at the OMN group at 72 h in comparison to the control and was higher in the ESPE groups compared to OMN groups. Furthermore, the *IL-1β* and *IL****-****6* levels in the OMN group at 72 h and in the ESPE groups were higher than the control one.

**Conclusion:**

The cytotoxic and genotoxic effect of ESPE on HGF is more significant than the OMN.

## Highlights

• Nano-composite treated groups revealed lesser cell viability than their controls.
• The ESPE groups and the OMN group at 72 h depicted higher total cell death.
• The ESPE groups and the OMN group at 72 h displayed higher levels of *Il-6* and *Il-1β*.

## Graphical abstract

**Figure:**
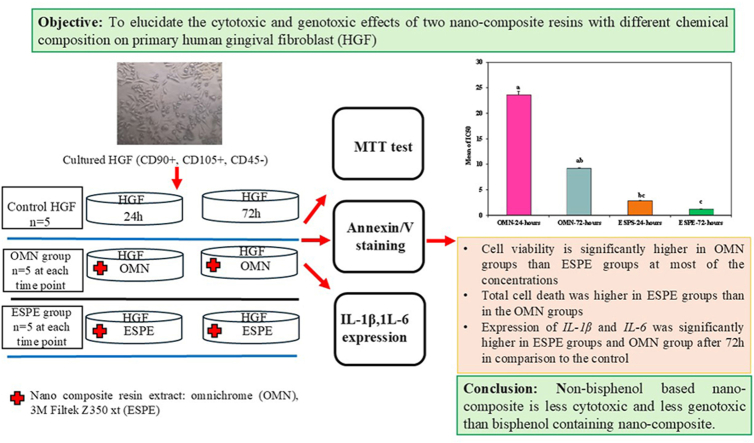

## Abbreviations

Bisphenol A Ethoxylated dimethacrylate: (Bis-EMA)
Bisphenol A-glycidyl Dimethacrylate: (Bis-GMA)
3-[4,5 dimethylthiazolyl-2-yl]-2,5-diphenyltetrazolium Bromide: (MTT)
Human gingival fibroblast cells: (HGFs)
Interleukin −6: (IL-6)
Interleukin–1β: (IL-1β)
3M Filtek Z350 xt composite resin: (ESPE)
3M Filtek Z350 xt extract treated HGFs: (ESPE group)
Omnichroma composite resin: (OMN)
Omnichroma extract treated HGFs: (the OMN group)
Tri Ethylene Glycol Di Methacrylate: (TEGDMA)
Urethane Di methacrylate: (UDMA)

## 1 Introduction

Human gingival fibroblast (HGFs) are the main gingival connective tissue cells and are crucial in producing and maintaining the extracellular matrix, the basic shape and function of the gingiva and in tissue repair and wound healing.^1^ In deep subgingival cavities, dental restorative materials are in contact with the HGFs, and composite resin has been shown to affect the viability of HGFs.^2^, ^3^, ^4^

Adequate restorative materials, including composite resin, should not be cytotoxic nor genotoxic to the surrounding oral tissues.^5^ Therefore, the biocompatibility of the dental restoratives in the oral cavity determines its success.^6^ Composite reins consist of various methacrylate monomers including bisphenol A-glycidyl methacrylate (Bis-GMA), triethylene glycol dimethacrylate (TEGDMA), and urethane dimethacrylate (UDMA),^7^ whereas the physical and mechanical properties of composite resin were largely improved by utilizing the nano-filler^2^**.**

The BIS-GMA is a commonly used monomer in composite resin restoration and contains the toxic component bisphenol A. The polymerization of the composite resin monomers in the organic matrix is incomplete and different unpolymerized monomer can diffuse and cause cytotoxic effect *in vitro* including: Bisphenol A Ethoxylated dimethacrylate (BIS-EMA), BIS-GMA, UDMA and TEGDMA.^8^ Previous studies revealed that components that are released from composite resin can enhance bacterial growth, oxidative stress, apoptosis and impact various cytokines' parameters in the oral tissue.^5^^,^^9^The cytokines are small glycoproteins that are crucial in immunity and inflammation^5^ and the HGF has an immunomodulatory capacity via the release of the inflammatory cytokines including interleukin-6 (IL-6) and interleukin 1β (IL-1β).^10^

The composite resin has been shown to be cytotoxic to human gingival fibroblasts and dental pulp stem cells.^2^, ^3^, ^4^^,^^11^ Although previous studies have investigated the cytotoxic effect of the commercially available nano-composite resins on the viability of the gingival cell lines^2^^,^^3^^,^^12^ and on primary gingival fibroblasts,^4^^,^^13^^,^^14^ the genotoxic effect of the nano-composite resin is hitherto in need for further investigations. Therefore, this study was designed to evaluate the comparative influence of two nano-composite resins with different chemical composition; bisphenol-based resin matrix composition and non-bisphenol based resin matrix composition on the *IL-1β* and *IL-6* inflammatory factors, and on the mode of cell death using annexin-V on HGF. Our null hypothesis was that the two nano-composite resins do not affect the expression of *IL-6*, *IL-1β* nor the annexin/V reaction in HGF.

## 2 Materials and methods

The experimental work was performed after getting the approval of Minia University's Faculty of Dentistry Research Ethics Committee (Ethical Code: RHDIRB2017122001, No: 85) and following the national guidelines and Declaration of Helsinki. An informed consent was obtained from the patient.

### 2.1 Isolation and culturing of HGFs

**Fig. 1:**
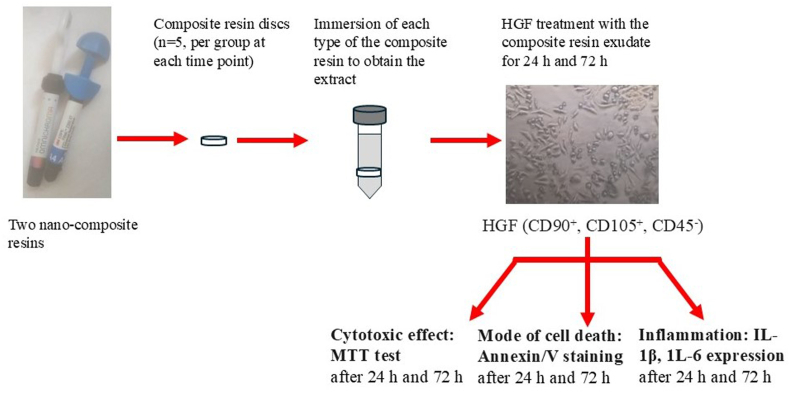
**The plan of the experiment.** The two nano-composite resins were molded into discs, soaked in culture media to obtain the extracts. The HGFs were exposed to the composite resin extracts for 24 h and 72 h, followed by assessment of HGF cytotoxicity, mode of cell death and inflammatory effect.

The gingival tissues were dissected from the same adult and healthy patient^15^ who underwent crown lengthening. The tissue sample was stored in sterile saline solution for 4 h followed by rinsing in phosphate buffered saline and cutting into small pieces. Thereafter, the tissues were enzymatically digested in dispase (2 mg/mL) and collagenase type 1A (2 mg/mL) (Sigma Aldrich, Saint Louis, MO, USA), harvested with (5 % CO2) for an hour at (37 °C),^4^ followed by filtering nylon mesh filter and centrifugation for 10 min at 1500 rpm. The isolated HGFs were cultured in (α-MEM) with 100 μg/mL Streptomycin and 1 % 100 unites/mL penicillin (Sigma Aldrich, Missouri, USA) (37 °C, 5 % CO2), the media was replaced every two days, till 80 % confluence. The HGF were trypsinized utilizing 0.25 % trypsin/0.02 % ethylenediaminetetraacetic acid. Dissociated cells were utilized in the following investigations^10^ (Fig. 1).

### 2.2 Characterization of gingival fibroblasts utilizing flow cytometer

The isolated HGF were recognized by their spindle shape and adherence and were further immunophenotyped as described previously in El-Badawy et al.^16^ by utilizing specific membrane markers; CD45^−^ FITC, CD105-FITC, and CD90-FITC (Thermo Fisher Scientific, USA) using flow cytometry multiparametric characterization. The cell count was adjusted to $10^{6}$/ml, suspended in phosphate-buffered saline, and followed by centrifugation for (10 min) at (800×*g*). The cellular pellets were rinsed in phosphate-buffered saline, and 5 μL of each antibody was added and harvested for (45 min) at (4 °C). At the end, the data were processed for flow cytometric analysis (Beckman Coulter, USA).^16^^,^^17^

### 2.3 Composite resin sample and extract preparation

A bisphenol-based composite resin matrix composition (3M Filtek Z350 xt (ESPE), 3M Oral Care; St Paul, MN, USA) and non-bisphenol based composite resin matrix composition (Omnichroma composite resin (OMN), Tokuyama Dental Corporation, Tokyo, Japan) were utilized. The OMN composite resin contains UDMA and TEGDMA monomers. The ESPE includes Bis-EMA, UDMA, TEGDMA, and Bis-GMA monomers.

A total 20 composite resin discs (n = 5 per group at each time point, for each nano-composite resin type)^14^ were prepared in line with the manufacturer's instructions at 2 mm in height and 5 mm in diameter, using molds. The samples were prepared in sterile conditions utilizing face mask and gloves by the operator. The uncured nanocomposite was added to the mold and was covered by a Mylar matrix strip to prevent oxygen inhibition. The unpolymerized resin was polymerized using LED curing light at intensity 720 mW/cm^2^ for 40 s (Celalux 3, High-Power LED curing-light; VOCO, Cuxaven, Germany).^4^ After setting of the nanocomposite resin, the discs was placed in a culture media with a ratio of surface area of the sample to the culture media volume at 117.8 mm^2^/ml according to the recommendations of ISO 10993-12.^2^ After being immersed for (48 h) at (37 °C), the sample extract solutions were filtered (Millipore; Billerica, MA, USA).^3^ The two nano-composite resin extracts were used in treating the HGFs in the following investigations (Fig. 1).

### 2.4 **The HGF viability detection using MTT test** (3-[4,5 dimethylthiazolyl-2-yl]-2,5-diphenyltetrazolium Bromide)

To assess the HGF's viability, MTT assay (Sigma, St. Louis, MO, USA) was utilized after 24 h and 72 h of exposure to composite resins' extracts. The assay is characterized by being simple, precise, rapid and widely used. The principle of the assay is that the MTT can be reduced by viable HGF mitochondrial dehydrogenase enzyme after being exposed to nanocomposite extracts. The enzyme modifies the yellowish tetrazolium salt to purplish formazan crystals.^18^^,^^19^ The HGFs were seeded in 96 plate (n = 5) at density of 2x10^5^/mL for (24 h) at (37 °C). The media was exchanged with fresh one, after which the HGFs were exposed to different dilutions of the two resin extracts (200, 100, 50, 25, 12.5, 6.25, 3.12, 1.56, 0.78, 0.39, 0.2). The HGFs were incubated and rinsed with phosphate-buffered saline to discard the dead cells. A 50 μl of MTT stock solution (0.5 mg/mL), were dispensed in every well. The plate was harvested at (37 °C) for (4 h) and the MTT solution was exchanged with DMSO (50 μl/well) for dissolution of the formazan crystals. Thereafter, an Elisa reader with a wavelength equal (570 nm) was utilized to measure light absorption (ELx-80, Bio-Tek Instruments, Inc, Winooski, VT, USA). The viable HGF percentage in each dilution was measured and the IC50 was determined.^20^

### 2.5 Annexin-V staining

The Annexin V/propidium iodide labelling (ab139418 propidium iodide flow cytometry kit/BD) and followed by flow cytometric analysis were used as it is a common and efficient methodology in differentiating between viable cells, early apoptotic, late apoptotic and necrotic cells and the method is based on the variation in the permeability of the plasma membrane in these cells. In addition, the propidium iodide is stable and economical nuclear stain. The annexin V has an affinity to the cell membrane phospholipid-phosphatidylserine- which is situated in the inner layer. At the early apoptotic event, the phosphatidylserine is repositioned to the outer membrane surface and bind to annexin V. Propidium iodide can enter the cells when their plasma and nuclear membranes are damaged. Therefore, HGF that were annexin V^−^/propidium iodide^−^, were sorted as viable cells. HGF which were annexin V^+/^propidium iodide^−^, were sorted as early apoptotic. HGF which were annexin V^+^/propidium iodide^+^, were sorted as necrotic/late apoptotic. HGF which were annexin V^−^/propidium iodide^+^, were sorted as necrotic ^(^^21^^,^ ^22^^)^. After incubation of HGFs with OMN and ESPE extracts for 24 h and 72 h, the HGFs were rinsed with phosphate-buffered saline for three times and centrifuged at (4 °C, 1500 rpm, 5 min) (Jouan Ki-21, France). The pellets were mixed with Annexin-HEPES buffer, thereafter (10 μl) of propidium iodide and (5 μl) of annexin-V were added and harvested for (15 min) in dark, at room temperature. The flow cytometric analysis was performed for propidium iodide at (585 nm) and for annexin-V/FITC at (488 nm) excitation (Becton-Dickinson, San Jose, CA, USA).^20^

### 2.6 Analysis of the level of expression of IL-6 and IL-1β utilizing RT-qPCR

To evaluate the inflammatory response of HGFs after the exposure to OMN and ESPE extracts for (24 h) and (72 h), the levels of IL-1β and IL-6 expression were assessed. After total RNA isolation utilizing RNeasy Mini Kit (Qiagen, Germantown, MD, USA), the RNA concentration was analyzed (Beckman dual spectrophotometer, Beckman Instruments, Ramsey, MN, USA). A cDNA reverse transcriptase kit was utilized to reverse transcribe the isolated RNA into cDNA (Applied Biosystems-Thermo Fischer Scientific, USA) followed by cDNA amplification (Sybr Green I PCR master kit, Thermo Fischer Scientific Inc., Lithuania).^20^ The utilized primers were: GAPDH Forward: 5′-GTCTCCTCTGACTTCAACAGCG-3′ and Reverse: 5′-ACCACCCTGTTGCTGTAGCCAA-3′, IL-6 Forward: 5′-TACCACTTCACAAGTCGGAGGC- 3′ and Reverse: 5′-CTGCAAGTGCATCATCGTTGTTC-3′, and IL-1β Forward: 5′-CCACAGACCTTCCAGGAGAATG-3′ and Reverse: 5′-GTGCAGTTCAGTGATCGTACAGG-3′.

### 2.7 Statistical analysis

A comparison of quantitative data was conducted using Kruskal Wallis test, then Post hoc test (Dunn's multiple comparison) utilizing IBM-SPSS ver. 20.0. (Armonk, NY, IBM Corp). When p-value is ≤ 0.05, the difference was set as significant.

## 3 Results

### 3.1 Immunophenotyping of HGFs

**Fig. 2:**
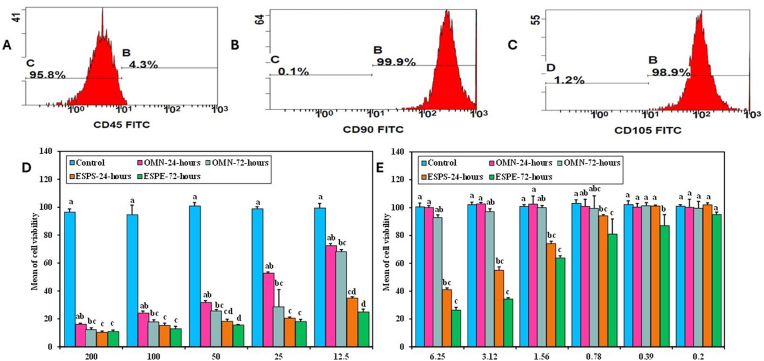
**Immunophenotyping of primary human gingival fibroblasts (HGFs) and lower HGF viability in the bisphenol-based composite resin (ESPE) groups compared to their controls**. Histograms representing the flow cytometric analysis of CD45 (A), CD90 (B), and CD105 (C) expression in HGF. (D, E) Viability percentage in HGF subjected to different concentrations of bisphenol based composite resin (ESPE) and non-bisphenol based composite resin (OMN) after 24 h and 72 h of exposure.

Characterization of the gingival isolated cells using flow cytometry revealed a pure isolation of HGFs and the cells were (CD105^+^/CD90^+^ and CD45^−^) (Fig. 2).

### 3.2 Higher cell viability in the OMN groups than the ESPE groups

The percentage of viable HGFs to the different concentration of OMN and ESPE extracts in relation to the untreated HGF was evaluated, following 24 h and 72 h of exposure to the resin extract (Table 1, Fig. 2). After 72 h of exposure to OMN extract, the percentages of viable HGFs were significantly lower at (200, 100, 50, 25 and, 12.5) concentrations, (p = 0.015, p = 0.024, p = 0.032, p = 0.01 and p = 0.032), respectively in comparison to the control. A significant cytotoxic change was obtained following 24 h of exposure to ESPE extract (200, 100, 50, 25, 12.5. 6.25 and 3.12) concentrations in comparison to the non-treated HGF (p < 0.001, p = 0.001. p = 0.001, p = 0.002, p = 0.001, p = 0.005 and p = 0.009), respectively (Fig. 2, Table 1). In addition, the cytotoxic effect was significant after a long term exposure to ESPE at 72 h at concentrations (200, 100, 50, 25, 12.5, 6.25, 3.12, 1.56) compared to the control (p = 0.001, p < 0.001, p < 0.001, p < 0.001, p < 0.001, p < 0.001, p < 0.001 and p = 0.001), respectively (Fig. 2, Table 1). An insignificant difference was observed in HGF's viability among control group and the OMN group after 24 h of exposure (Table 1, Fig. 2). After 24 h as well as after 72 h of exposure of HGF to the two composite resin extracts, a significant difference was observed between the IC50 of OMN (23.60, 9.21) and ESPE (2.83, 1.22) groups, respectively (p = 0.008).

**Table 1:** Comparison between the different studied groups according to cell viability.

	Control	OMN-24-h	OMN-72-h	ESPS-24-h	ESPE-72-h	H	p
200							
Min. – Max.	92.85–98.97	15.57–17.24	10.56–14.18	9.17–11.68	9.73–12.23	20.579	<0.001∗
Mean ± SD.	96.40 ± 2.32	16.19 ± 0.69	12.30 ± 1.34	10.36 ± 0.91	10.91 ± 1.07		
Median (IQR)	96.40^a^(95.9–97.9)	16.19^ab^(15.6–16.4)	12.30^bc^(11.7–12.8)	10.36^c^(10.0–10.6)	10.91^c^(10.0–11.7)		
**100**							
Min. – Max.	82.84–100.6	21.96–25.58	16.12–20.02	13.07–17.24	10.01–14.46	22.144∗	<0.001∗
Mean ± SD.	94.59 ± 6.92	24.19 ± 1.35	17.86 ± 1.42	15.29 ± 1.63	12.93 ± 1.76		
Median (IQR)	96.74^a^(94.6–98.1)	24.46^ab^(24.2–24.7)	17.86^bc^(17.2–18.1)	15.29^c^(14.5–16.4)	13.07^c^(12.9–14.2)		
**50**							
Min. – Max.	96.74–103.4	30.02–33.64	24.46–26.97	16.12–20.57	15.01–16.12	22.995∗	<0.001∗
Mean ± SD.	100.8 ± 2.48	31.76 ± 1.34	25.65 ± 1.07	18.21 ± 1.59	15.57 ± 0.44		
Median (IQR)	101.5^a^ (100.9–101.8)	31.76^ab^ (31.1–32.3)	25.65^bc^ (24.7–26.4)	18.21^cd^ (17.8–18.4)	15.57^d^ (15.3–15.9)		
**25**							
Min. – Max.	97.02–100.9	51.99–54.21	7.23–36.70	19.18–21.41	15.85–20.02	20.684∗	<0.001∗
Mean ± SD.	98.83 ± 1.50	52.75 ± 0.87	28.63 ± 12.38	20.50 ± 0.82	18.07 ± 1.56		
Median (IQR)	98.83^a^(97.9–99.5)	52.54^ab^(52.3–52.8)	34.75^bc^(28.6–35.9)	20.57^c^(20.5–20.9)	18.07^c^(17.5–18.9)		
**12.5**							
Min. – Max.	93.96–102.6	70.89–74.23	66.44–70.06	33.92–36.42	21.96–26.97	23.077∗	<0.001∗
Mean ± SD.	99.39 ± 3.36	72.42 ± 1.42	68.18 ± 1.41	34.82 ± 0.97	24.95 ± 2.04		
Median (IQR)	99.39^a^ (99.3–101.8)	72.42^ab^ (71.2–73.4)	68.18^bc^ (67.3–68.9)	34.75^cd^ (34.2–34.8)	24.95^d^ (24.2–26.7)		
**6.25**							
Min. – Max.	97.02–104.0	98.69–102.0	89.52–94.80	39.48–42.81	24.46–29.47	21.998∗	<0.001∗
Mean ± SD.	100.6 ± 2.69	100.1 ± 1.35	92.78 ± 1.98	41.14 ± 1.19	26.34 ± 2.0		
Median (IQR)	100.6^a^ (99.3–102.3)	100.1^a^ (99.0–100.6)	93.13^ab^ (92.8–93.7)	41.14^bc^ (40.9–41.4)	26.34^c^ (24.7–26.7)		
**3.12**							
Min. – Max.	99.52–104.8	101.5–104.0	93.69–98.97	52.26–58.94	33.08–35.31	21.961∗	<0.001∗
Mean ± SD.	102.1 ± 1.88	102.7 ± 1.07	97.09 ± 2.10	55.04 ± 2.43	34.33 ± 0.89		
Median (IQR)	102.1^a^ (101.8–102.3)	102.7^a^ (101.8–103.4)	97.09^ab^ (97.0–98.7)	54.77^bc^ (54.2–55.0)	34.33^c^ (33.9–35.0)		
**1.56**							
Min. – Max.	99.52–102.6	96.74–111.5	97.86–101.8	71.72–76.45	61.44–65.33	18.755∗	0.001∗
Mean ± SD.	100.9 ± 1.29	102.5 ± 5.86	100.0 ± 1.55	74.16 ± 1.72	63.87 ± 1.54		
Median (IQR)	100.9^a^ (99.8–101.8)	102.5^a^ (97.9–104.0)	100.0^ab^ (99.3–101.2)	74.16^bc^ (73.7–74.8)	63.87^c^ (63.7–65.1)		
**0.78**							
Min. – Max.	99.25–106.2	93.13–107.6	90.35–114.3	93.13–95.08	73.67–99.80	12.693∗	0.013∗
Mean ± SD.	103.1 ± 2.55	100.9 ± 5.15	99.39 ± 9.12	94.10 ± 0.85	80.97 ± 10.89		
Median (IQR)	103.1^a^ (102.6–104.3)	101.2^ab^ (100.9–101.8)	98.97^abc^ (94.0–99.4)	94.10^bc^ (93.4–94.8)	75.62^c^ (74.8–81.0)		
**0.39**							
Min. – Max.	99.80–106.8	96.74–104.0	98.97–105.1	99.80–101.8	78.95–96.47	12.628∗	0.013∗
Mean ± SD.	102.2 ± 2.70	100.4 ± 2.65	101.3 ± 2.34	101.1 ± 0.75	87.01 ± 7.88		
Median (IQR)	101.8^a^ (100.6–102.2)	100.4^a^ (99.5–101.5)	101.2^a^ (99.8–101.3)	101.2^a^ (101.1–101.5)	87.01^b^ (79.5–93.1)		
**0.2**							
Min. – Max.	99.52–102.6	93.13–109.0	93.13–105.9	99.80–104.0	93.13–97.86	9.126	0.058
Mean ± SD.	101.0 ± 1.10	100.2 ± 5.89	99.59 ± 4.89	102.0 ± 1.51	94.94 ± 1.83		
Median (IQR)	101.0^a^ (100.6–101.2)	100.2^a^ (97.0–101.5)	99.59^a^ (97.0–102.3)	102.0^a^ (101.8–102.6)	94.94^a^ (93.7–95.1)		

5 replica for each group IQR: **Inter quartile range**.

H: H for **Kruskal Wallis test,** Pairwise comparison bet. each 2 groups was done using **Post Hoc Test (Dunn's for multiple comparisons test)**.

p: p value for comparing between the studied groups.

∗: Statistically significant at p ≤ 0.05.

Median with **any Common letter ^(a-c)^** are not significant (**OR** Median with **totally Different letters ^(a-c)^** are significant).

Comparing the viability results of OMN and ESPE groups revealed higher viability in OMN groups than in ESPE groups after exposure at both 24 h and 72 h at most of the concentrations (Table 1, Fig. 2). In addition, the cell viability percentage in both groups was significantly decreased by increasing the concentration (inverse relationship) (Table 1, Fig. 2).

### 3.3 Higher total cell death in ESPE groups after 24 h and 72 h of exposure to the resins'extract compared to the control

**Fig. 3:**
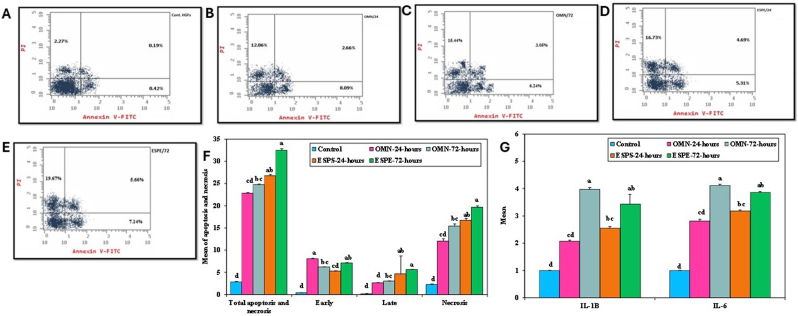
**Higher total cell death and pre-inflammatory mediators' expression in human gingival fibroblasts (HGFs) treated with bisphenol based composite resin (ESPE) compared to their controls**. Flow cytometric analysis of primary human gingival fibroblasts (HGF) treated with non-bisphenol based composite (OMN) and bisphenol based composite (ESPE), utilizing annexin V/propidium iodide (PI) in control group (A), OMN group after 24h of exposure to OMN extract (B), 72h (C), ESPE extract for 24h (D) and 72h (G). (F) Bar graph representing annexin V/PI results utilizing flow cytometry. (G) The *IL-1β* and *IL-6* levels in HGF after exposure to ESPE and (OMN) after 24h and 72h.

The exposure of HGF to OMN or ESPE extracts for 24 h or 72 h revealed increased total apoptosis and necrosis after exposure to OMN at 72 h (p = 0.032), ESPE at 24 h (p = 0.001), and 72 h (p < 0.001) in comparison to the control. Furthermore, the total apoptosis and necrosis were significantly lower in OMN groups in comparison to ESPE groups after 24 h (p = 0.032) and 72 h (p = 0.032). The HGF revealed significantly increased early apoptosis after exposure to OMN after 24 h (p < 0.001), OMN after 72 h (p = 0.032) and ESPE after 24 h (p = 0.001) in comparison to the control. The late apoptosis in the HGF was significantly increased after exposure to OMN extract to 72 h (p = 0.032), ESPE extract to 24 h (p = 0.001), and ESPE extract to 72 h (p < 0.001) in comparison to the control. Exposure to OMN extract at 72 h, to ESPE extracts at 24 h and at 72 h resulted in increased necrosis in HGF (p = 0.030) and (p = 0.001), respectively compared to the untreated HGF. Late cell apoptosis and necrosis were significantly higher in ESPE groups after 24 h and 72 h of extracts exposure compared to OMN groups following 24 h and 72 h of extract exposure (p = 0.032 and p = 0.033), respectively (Fig. 3, Table 2).

**Table 2:** Comparison between the different studied groups according to mode of cell death.

Apoptosis and necrosis	Control	OMN-24-h	OMN-72-h	ESPS-24-h	ESPE-72-h	H	p
Total apoptosis and necrosis							
Min. – Max.	2.70–2.99	22.61–22.96	24.56–24.90	26.48–27.21	32.05–33.01	23.086∗	<0.001∗
Mean ± SD.	2.88 ± 0.11	22.82 ± 0.13	24.73 ± 0.14	26.73 ± 0.28	32.47 ± 0.35		
Median (IQR)	2.90^d^(2.9–2.9)	22.81^cd^(22.8–22.9)	24.73^bc^(24.6–24.8)	26.63^ab^(26.6–26.7)	32.40^a^(32.4–32.5)		
**Early**							
Min. – Max.	0.40–0.44	8.01–8.19	6.22–6.26	5.24–5.41	7.08–7.17	23.086∗	<0.001∗
Mean ± SD.	0.42 ± 0.02	8.09 ± 0.06	6.24 ± 0.02	5.31 ± 0.07	7.14 ± 0.04		
Median (IQR)	0.42^d^(0.41–0.43)	8.09^a^(8.1–8.1)	6.24^bc^(6.2–6.3)	5.31^cd^(5.3–5.3)	7.15^ab^(7.1–7.2)		
**Late**							
Min. – Max.	0.10–0.24	2.60–2.72	3.01–3.08	4.65–4.75	5.61–5.71	23.086∗	<0.001∗
Mean ± SD.	0.19 ± 0.05	2.66 ± 0.06	3.05 ± 0.03	4.69 ± 0.04	5.66 ± 0.04		
Median (IQR)	0.20^d^(0.19–0.22)	2.66^d^(2.6–2.7)	3.05^bc^(3.05–3.06)	4.69^ab^(4.66–4.70)	5.66^a^(5.6–5.7)		
**Necrosis**							
Min. – Max.	2.10–2.40	11.50–12.80	15.0–16.20	16.20–17.30	19.38–20.10	22.995∗	<0.001∗
Mean ± SD.	2.27 ± 0.11	12.06 ± 0.52	15.44 ± 0.48	16.73 ± 0.39	19.67 ± 0.30		
Median (IQR)	2.28^d^(2.3–2.3)	12.06^cd^(11.6–12.3)	15.44^bc^(15.1–15.5)	16.73^ab^(16.6–16.8)	19.67^a^(19.4–19.8)		

5 replica for each group IQR: **Inter quartile range**.

H: H for **Kruskal Wallis test,** Pairwise comparison bet. each 2 groups was done using **Post Hoc Test (Dunn's for multiple comparisons test)**.

p: p value for comparing between the studied groups.

∗: Statistically significant at p ≤ 0.05.

Median with **any Common letter ^(a-d)^** are not significant (**OR** Median with **totally Different letters ^(a-d)^** are significant).

### 3.4 Upregulation of *IL-1β* and *IL-6* in ESPE groups after short and long nano-composite extract exposure periods

Evaluation of the cytokines (*IL-1β* and *IL-6*) in HGF after exposure to OMN or ESPE extracts to 24 h or 72 h revealed a significant upregulation of *IL-6* and *IL-1β* in OMN group after 72 h (p < 0.001) and ESPE groups following 24 h (p = 0.031) and after 72 h (p = 0.001), in comparison to the non-treated HGF. The expression of *IL-6* and *IL-1β* in the OMN group after 24 h was significantly different from that at 72 h of extract exposure (p = 0.002) and (p = 0.001), respectively (Fig. 3, Table 3).

**Table 3:** Comparison between the different studied groups according to IL-1β and IL-6.

	Control	OMN-24-h	OMN-72-h	ESPS-24-h	ESPE-72-h	H	p
IL-1B							
Min. – Max.	1.0–1.0	2.01–2.11	3.90–4.05	2.50–2.64	3.03–4.01	22.773∗	<0.001∗
Mean ± SD.	1.0 ± 0.0	2.07 ± 0.04	3.97 ± 0.07	2.55 ± 0.06	3.43 ± 0.36		
Median (IQR)	1.0^d^(1.0–1.0)	2.07^cd^(2.06–2.10)	3.97^a^(3.9–4.0)	2.55^bc^(2.5–2.6)	3.43^ab^(3.2–3.4)		
**IL-6**							
Min. – Max.	1.0–1.0	2.71–2.90	4.03–4.16	3.14–3.22	3.81–3.90	23.265∗	<0.001∗
Mean ± SD.	1.0 ± 0.0	2.81 ± 0.07	4.11 ± 0.05	3.18 ± 0.04	3.86 ± 0.03		
Median (IQR)	1.0^d^(1.0–1.0)	2.81^cd^(2.8–2.8)	4.11^a^(4.1–4.2)	3.18^bc^(3.2–3.2)	3.86^ab^(3.86–3.87)		

5 replica for each group IQR: **Inter quartile range**.

H: H for **Kruskal Wallis test,** Pairwise comparison bet. each 2 groups was done using **Post Hoc Test (Dunn's for multiple comparisons test)**.

p: p value for comparing between the studied groups.

∗: Statistically significant at p ≤ 0.05.

Median with **any Common letter ^(a-d)^** are not significant (**OR** Median with **totally Different letters ^(a-d)^** are significant).

## 4 Discussion

In the present work, the cytotoxic and the inflammatory effects, in addition to the mode of cell death, on HGFs in response to exposure to OMN and ESPE nano-composite resins' extracts were evaluated. The HGFs were selected for their ease of isolation, culturing and for their clinical relevance.^3^^,^^23^ This is in agreement with previous work.^2^, ^3^, ^4^^,^^12^, ^13^, ^14^ Previous studies reported that the gingival epithelium is unavoidably injured in deep subgingival cavity with the consequent exposure of the gingival lamina propria and the inevitable contact of the resin restoration with the gingival fibroblasts. These gingival fibroblasts play a crucial role in the subsequent healing process.^23^

The ISO 10993-5 specified that a material is potentially cytotoxic if the measurable viability is less than 70 % of the negative control.^2^^,^^24^^,^^25^ In the current study, the viability of HGFs after being exposed to OMN versus ESPE extracts was evaluated. The viability percentage decreased in OMN and ESPE groups as the concentration increased compared to the controls. At most of the concentration, the viability percentages in the OMN group at 24 h and 72 h were higher than that at the ESPE group. Our findings are in line with the data of Ilie et al., Duzyol et al., and Beltrami et al.^2^^,^^3^^,^^9^ on OMN and the data of Tamilselvam et al. and Gonçalves et al. on ESPE.^26^^,^^27^ Previous studies suggested that the decrease in the viability of HGFs after restoration with composite resin is due to the presence of un-bonded monomers that were released during and after the polymerization process due to the incomplete conversion of all monomers into polymers.^28^^,^^29^ In addition, the difference in biocompatibility between ESPE and OMN groups could be due to the difference in the chemical composition of the two composite resins as OMN consists of two types of monomers (TEGDMA and UDMA), but ESPE contains 4 types of monomers (TEGDMA, UDMA, Bis-GMA, Bis-EMA).^2^^,^^30^^,^^31^ Furthermore, previous work on HGFs revealed that UDMA is less toxic than the Bis-GMA,^23^ BIS-EMA mimics TEGDMA in the cytotoxic effect,^8^ and that BisGMA is the most cytotoxic monomer followed by UDMA and TEGDMA.^7^^,^^12^

The inflammatory process is accompanied by the secretion of a large number of mediators, such as proteolytic enzymes, immunoregulatory factors, and cytokines.^32^ It has been reported that residual monomer led to gingival irritation and inflammation.^33^ In the present work, the expression of *IL-1β* and *IL-6* were upregulated in HGFs after exposure to OMN at 72 h, ESPE at 24 h and ESPE at 72 h in comparison to the control group. Our findings are in accordance with the data of Duzyol et al.,^9^ who demonstrated an increased expression of *IL-6* and *IL-1β* in mouse fibroblast cell line upon exposure to composite resin.^9^ The upregulation of *IL-6* has also been shown in HGF upon exposure to BIS-GMA based composite and UDMA based composite resins.^23^ Furthermore, increased *IL-6* was detected in HGFs treated with a methacrylate material extract. In addition, endodontic sealers containing UDMA upregulated *IL-6* in osteoblasts, pulp cells and periodontal ligament cells.^34^^,^^35^ Also, subgingival class V restoration utilizing composite resin containing methacrylate monomers caused upregulation of *IL-6* in the gingival crevicular fluid.^5^

In the present study, the total apoptotic cell percentage and necrotic cell percentage were higher in OMN group after exposure for 72h and in ESPE group following 24 h and 72 h of exposure compared to the non-treated HGF. The late apoptotic cell death and the cell necrosis were significantly lower in OMN group at 24 h and 72 h compared to ESPE at 24 h and 72 h, respectively. These data confirm the relevance to the cytotoxic behavior of the two nano composite resins under study. Our data are in agreement with previous work of Fujioka-Kobayashi et al.^23^ on HGFs who demonstrated that the bisphenol based and UDMA based composite upregulated apoptotic markers in HGFs.^23^ The authors reported that apoptosis was induced in different cell types by the resin monomer via disturbing the reactive oxygen species which leads to damage to the DNA and followed by apoptosis.^36^

The limitation of this study was the absence of the in vivo effect of the two nano-composite resins on HGFs. In addition, future studies evaluating nanocomposite chemical composition, dose, time of exposure, cellular membrane integrity, cell volume, refractive index and cytoplasm volume are needed. Furthermore, more studies are needed to evaluate the effect of each single monomer of the nano-composites on HGF as well as the individual effect of each type of their nanoparticles. Further investigations should study the other molecular changes in response to the different composition of nano-composite resins on HGF. Another limitation of the current work is the small sample size. Further studies addressing additional genetic effect of nano-composite on HGF with larger sample size are required.

In conclusion, the biological behavior of HGFs in response to exposure to bisphenol containing and non-bisphenol containing composite resin extracts may depend on the material composition. Although both resin extracts have cytotoxic effects and have influence on the mode of the cell death and the inflammatory response of the HGFs, the least biological effect was depicted in the non-bisphenol-based composite resin. Therefore, the local inflammatory response after restoration of subgingival dental cavities with bisphenol-based composite resin might be due to the increase in IL-1β and IL-6 and the apoptotic/necrotic influence of the resin on the gingival fibroblasts.

**Clinical relevance**: The cytotoxic and genotoxic effects were more significant in conditions where the bisphenol-based composite resin extract was in contact with HGFs than with non-bisphenol-based resin. Therefore, more significant cytotoxic effect, apoptosis and necrosis as well as inflammation induction are expected to occur in HGFs after bisphenol-based resin placement in subgingival cavities with traumatized gingival tissues than with non-bisphenol based composite, and consequently interfering with the local tissue healing. Thus, to minimize the local adverse effects when restoring subgingival cavities, it is advisable to use non-bisphenol based composite to obtain the least cellular response.

## Funding

This research did not receive any specific grant from funding agencies in the public, commercial or not-for-profit sectors.

## Declaration of competing interest

None. We wish to confirm that there are no known conflicts of interest associated with this publication and there has been no significant financial support for this work that could have influenced its outcome.

## Data Availability

All data are available from the corresponding author upon a reasonable request.
